# Evaluation of GPT-5 in Periodontitis Staging and Grading: Retrospective Observational Study

**DOI:** 10.2196/88407

**Published:** 2026-04-06

**Authors:** Ihunna Amugo, Katie Lee Frederickson, Harshana Rajakaruna, Hua Xie, Pandu Gangula, Anil Shanker, Qingguo Wang

**Affiliations:** 1 Department of Oral Diagnostic Sciences (ODS) & Research, School of Dentistry, Meharry Medical College, Nashville, TN, 37208, United States, 1 615-327-6786; 2 Department of Biochemistry, Cancer Biology, Neurosciences and Pharmacology, School of Medicine, Meharry Medical College, 1023 21st Ave N, Nashville, TN, 37208, United States; 3 The Office for Research and Innovation, Meharry Medical College, Nashville, TN, United States

**Keywords:** large language model, ChatGPT, GPT-5, dental care, dental education, periodontitis, periodontitis staging and grading

## Abstract

**Background:**

Periodontitis is a chronic gum disease affecting approximately 42% of adults aged 30 years and older in the United States. Training dental students to accurately diagnose and manage periodontitis is a critical component of dental education and clinical care. Recent advances in large language models offer new opportunities to support both domains, yet their performance in periodontal diagnosis remains largely unexplored, particularly for newer models such as GPT-5.

**Objective:**

This study conducted an exploratory evaluation of GPT-5’s ability to stage and grade periodontitis.

**Methods:**

A total of 25 publicly available clinical cases explicitly reporting periodontitis stage and grade were identified through Google and PubMed searches. Each case description was entered into GPT-5 using a zero-shot prompting approach to assess guideline-based reasoning without exemplar conditioning. The model’s predictions were compared with the published reference diagnoses. Performance was measured using accuracy, 95% CI, unweighted Cohen κ, and weighted Cohen κ.

**Results:**

Across these cases, GPT-5 showed marked class-dependent performance and a tendency to overestimate disease severity. Grading performance was notably imbalanced, with high recall for grade C but substantially lower discrimination for grade B. GPT-5 achieved a staging accuracy of 68% (95% CI 48.4%-82.8%) and a grading accuracy of 77.3% (95% CI 56.6%-89.9%), with corresponding Cohen κ values of 0.454 (95% CI 11.0%-75.6%) and 0.179 (95% CI −15.8% to 63.8%), respectively. While staging performance showed fair agreement beyond chance, the low κ for grading indicates poor agreement and limited reliability in distinguishing periodontal disease severity.

**Conclusions:**

These findings suggest that although GPT-5 demonstrates potential for guideline-based periodontitis staging and grading, its current diagnostic performance, particularly for periodontitis grading, limits its use in clinical assessment and educational training. Meaningful application in periodontal diagnosis and training will require substantial improvements in reliability and rigorous validation in larger, more diverse, and prospectively collected datasets.

## Introduction

As the demand for accessible, accurate, and cost-effective resources to support dental care and education continues to grow, large language model (LLM)–based chatbots, such as ChatGPT [1], have emerged as potentially useful tools. Although not originally developed for dental and health care applications, these systems can generate humanlike responses with remarkable accuracy on many health-related topics [2-8], offering new opportunities for disease surveillance, biomedical research, and education.

Compared with traditional resources, LLMs offer distinct advantages for education and diagnostic support, including lower cost, continuous availability without the need for appointments, good accuracy for many diseases and conditions, customizable interactions, and user-friendly interfaces. As a result, people increasingly turn to them for medication information, self-diagnosis, and disease prevention guidance [9-12]. Clinicians, dental students, and medical students also use them to acquire knowledge and support clinical decision-making [13,14].

A growing body of research has examined LLMs’ use in dental care and education [5,15-18]. Several benchmark studies have demonstrated LLMs’ competitive performance on the American Academy of Periodontology in-service examination [6], the United States Medical Licensing Examination [2,7], and other major assessments [1,3,8]. In an educational study involving 77 second-year dental students, those who used LLMs for learning assignments were found to perform better on knowledge examinations than peers relying on traditional methods [19]. Furthermore, Rahad et al [5] showed that ChatGPT excelled in recognizing and correcting specialized dental terminology and achieved 66.7% accuracy in extracting and synthesizing information from documents. In the clinical context, Tastan Eroglu et al [4] evaluated ChatGPT-3.5 on 200 untreated periodontitis cases and reported moderate performance for staging and grading.

Despite these advances, critical gaps remain. Most prior studies used nonpublic datasets, limiting the reproducibility of their findings. Moreover, given the continual iteration and rapid improvement of LLMs, earlier assessments may not accurately reflect the capabilities of newer models such as GPT-5, released in August 2025, whose performance in dentistry has not yet been systematically evaluated. Evaluation of LLMs in high-stakes dental clinical contexts is essential to establish quality control mechanisms, mitigate risks of inaccurate or biased outputs, and guide their safe adoption into dental education and care.

An important component of dental education and care is training students to diagnose and manage periodontitis, a chronic gum disease affecting approximately 42% of adults aged 30 years and older in the United States [20]. Periodontitis staging (I-IV) reflects disease severity and extent based on levels of destroyed tissues, including gingival attachment and alveolar bone, while grading (A-C) estimates the rate of progression and future risk [21-23]. Even with explicit and standardized criteria for staging and grading [21-23], clinical diagnosis of periodontitis remains challenging and context dependent, requiring careful integration of radiographic evidence, periodontal charting, and patient-specific risk factors. An LLM capable of accurately analyzing, staging, and grading clinical periodontitis cases could serve as both a valuable diagnostic aid for clinicians and a useful educational resource for students. To address this need, we conducted an exploratory evaluation of the performance of the newly released GPT-5 in staging and grading periodontitis cases.

## Methods

### Case Identification and Data Collection

This study was conducted as an exploratory, case-based evaluation of GPT-5’s diagnostic performance in periodontitis staging and grading. The unit of analysis was publicly available published clinical case descriptions rather than individual patients; thus, no clinical intervention or participant recruitment was involved. Standardized textual prompts were submitted to GPT-5, and the resulting categorical outputs were compared against the reference diagnoses reported in the source publications. The scope was strictly limited to assessing model-level diagnostic agreement and did not include evaluation of uncertainty calibration, triage behavior, clinical implementation, feasibility, or user acceptability.

We identified dental clinical cases by searching Google and PubMed in August and September 2025 using the keywords “periodontitis,” “staging,” and “grading.” PubMed processes such queries through its automatic term mapping system, which searches across Medical Subject Headings (MeSH) terms and text fields. In contrast, Google search results are dynamic, not field restricted, and were accordingly used to supplement PubMed retrieval. These searches targeted peer-reviewed articles, case reports, and publicly available teaching materials that explicitly described periodontitis staging and grading according to established clinical criteria.

**Figure 1.**
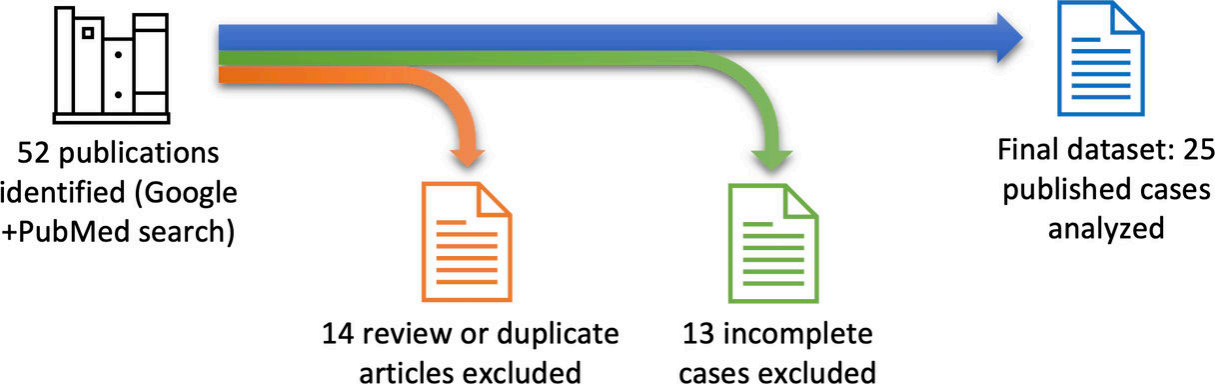
Data collection and case selection workflow.

All records retrieved from the searches were screened manually. Review articles; duplicate records; and reports lacking complete staging or grading diagnoses, sufficient periodontal clinical descriptions, or adequate medical and dental histories were excluded. Records that did not provide sufficient quantitative or descriptive information to independently determine stage and grade under the 2017 World Workshop criteria were also excluded. After screening, 25 (48%) cases were retained from a total of 52 identified records. The workflow for data collection and case selection is illustrated in Figure 1.

Most of the public cases collected included panoramic and periapical radiographs, which, together with patients’ dental histories and periodontal charting, play a central role in diagnosis. In these cases, the radiographs had already been systematically evaluated by the original authors, and numerical measures of the bone loss were extracted and reported in their publications. We manually extracted these clinically meaningful data from the publications and used them directly in our assessments. Radiographic images themselves were not used in our analyses.

It is important to note that publicly available and teaching-oriented cases, which are often unusually well documented and may not fully reflect the spectrum of routine clinical presentations, may introduce selection bias and limit generalizability. As a result, the GPT-5 performance observed in this exploratory study should be interpreted cautiously and may not reflect real-world diagnostic performance. These limitations are discussed in greater detail in the Discussion section.

### GPT-5 Prompting and Evaluation

While GPT-5 can process multimodal data, we used only its interactive textual interface. All analyses were conducted between September 1 and 12, 2025, using the free GPT-5 version, without any paid enhancements. GPT-5 was accessed through its publicly available interactive interface, which does not permit user control over system-level parameters such as temperature and system prompts. Therefore, all interactions were conducted using the default system configuration (temperature=1.0).

Given GPT-5’s demonstrated accuracy and reasoning improvements over earlier models [24], and because the criteria for periodontitis staging and grading outlined in the 2017 World Workshop are strictly guideline based [21-23], we adopted a zero-shot prompting strategy. This approach evaluates GPT-5’s ability to apply explicit clinical thresholds and decision logic without exemplar conditioning. In contrast to few-shot prompting or fine-tuning [25-27], which may introduce anchoring effects or label leakage, zero-shot prompting provides a more conservative and transparent assessment of model reasoning. This design was intentionally selected to evaluate GPT-5’s intrinsic application of guideline-based criteria without exemplar-based calibration.

Using zero-shot prompting, case descriptions were submitted directly to the model without examples or prior instructions, and GPT-5 was asked to return predictions of periodontitis stage and grade. Categorical outputs were recorded exactly as generated, and diagnostic reasoning text was not scored. Each case was processed independently, with no feedback incorporated across cases. Before submission, case descriptions were lightly reformatted to correct line breaks and formatting artifacts introduced during PDF extraction to improve readability. No clinical content was added, removed, reworded, or reorganized. An example prompt and model response are provided in Multimedia Appendix 1.

To assess response stability, each case was tested in multiple sessions using slight variations in prompt phrasing (eg, “Can you help determine the periodontitis stage and grade of this patient?” or “Please stage and grade the periodontitis of this patient”). The model consistently generated identical predictions, regardless of prompt phrasing or session timing, indicating stable behavior at the decision level under these conditions. For the final analyses reported in this study, we used a standardized prompt, “Please stage and grade the periodontitis of this patient,” followed by the corresponding clinical case description.

Data collection and analysis were conducted by 7 domain experts, all of whom are coauthors of this study. The team included a dental student, a medical student, a mathematician, 2 professors from the School of Dentistry, and 2 professors from the School of Medicine at Meharry Medical College. Their responsibilities included extracting clinical cases from publications, developing prompts for GPT-5, and verifying the model’s responses.

### Performance Metrics

GPT-5 predictions were compared against published ground-truth diagnoses reported in the original case sources. The primary outcomes were staging accuracy, grading accuracy, and agreement beyond chance, quantified using unweighted Cohen κ. Because staging (I-IV) and grading (A-C) represent ordinal categories, weighted Cohen κ was additionally calculated using the R package irr (version 0.84.1) to account for partial agreement across adjacent categories.

We calculated 95% CIs for accuracy (Wilson method) and κ statistics (bootstrap resampling, 2000 iterations). To address potential class imbalance, macro-averaged *F*_1_-scores and balanced accuracy were computed. In addition, confusion matrices were analyzed to characterize error patterns, including adjacent-category misclassification and directional bias (overestimation vs underestimation). All data analyses were conducted in R (version 4.3.3), and visualizations were generated using the R package ggplot2 (version 3.5.1).

### Ethical Considerations

This study used publicly available, published clinical cases and did not involve human or access to protected health information. Only deidentified textual case descriptions were submitted to GPT-5; no patient images, radiographs, protected health information, or identifiable data were entered into or transmitted through the external LLM platform. Case materials were reviewed before submission to confirm the absence of identifiable information. Because this study involved only publicly available, deidentified materials and did not involve human participants, it does not constitute human subjects research and, therefore, does not require institutional review board approval. Accordingly, informed consent was not required and participant compensation is not applicable. Prompts and outputs were stored on secure, password-protected institutional computers accessible only to the research team. This study was designed and reported according to the Standards for Reporting Diagnostic Accuracy (STARD) and STARD-AI principles [28,29], and a completed checklist is provided in Checklist 1.

## Results

### Description of Periodontitis Cases

Of the 25 periodontitis cases collected for evaluating GPT-5, 2 (8%) were borderline cases with staging ambiguities between stage III and stage IV, and 3 (12%) provided only staging information in the original publications. Full case descriptions and corresponding sources are provided in Table S1 in Multimedia Appendix 1. The median age of these patients was 45 (IQR 35-56) years, with most cases (17/25, 68%) occurring between 35 and 64 years. There were 3 participants aged 5-19 years, 4 aged 20-34 years, 9 aged 35-49 years, 8 aged 50-64 years, and 1 aged 65-80 years. Female participants comprised the majority (n=18, 72%), while male participants accounted for 28% (n=7) of the cohort. In terms of disease severity, stage III periodontitis was most common (n=14, 56%), followed by stage IV (n=9, 36%), stage II (n=1, 4%), and stage I (n=1, 4%). For grading, most cases were classified as grade C (n=17, 77%), with smaller proportions in grade B (n=5, 23%).

### Workflow for Evaluating GPT-5

**Figure 2.**
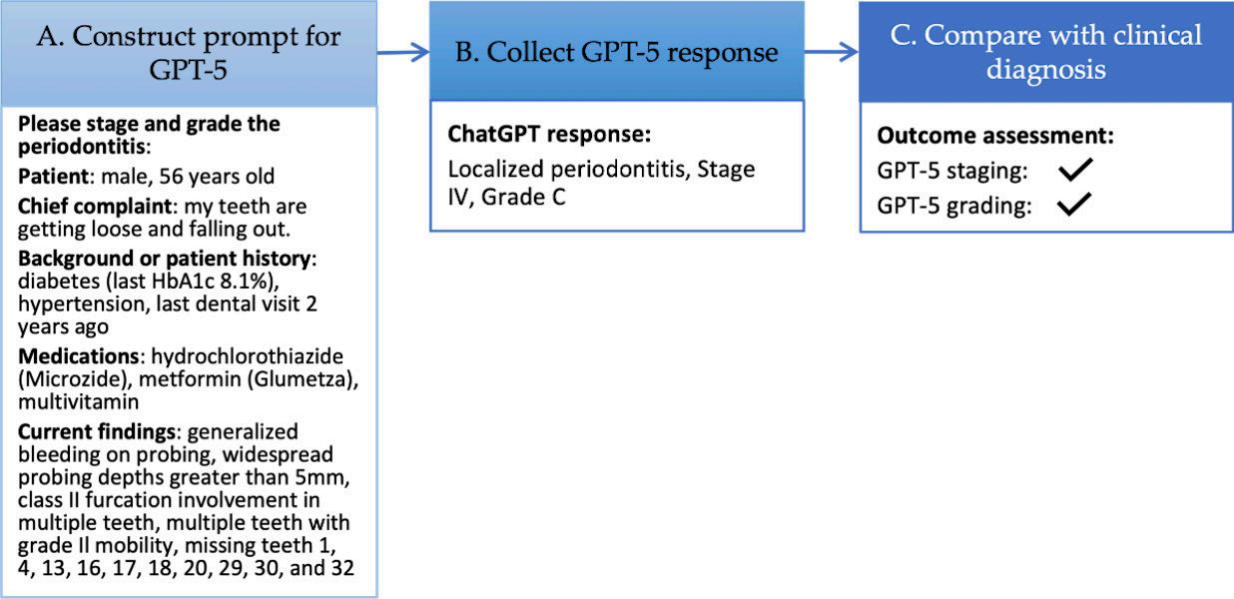
Framework for evaluating GPT-5 performance in periodontitis staging and grading. (A) A prompt was constructed from a publicly available case [30], with minor formatting adjustments (eg, line breaks and spacing) to improve readability for the model while preserving the original content. (B) GPT-5 generated categorical outputs for periodontitis stage (I-IV) and grade (A-C). (C) Model predictions were compared with the clinical reference diagnoses reported in the original publication to assess accuracy.

Figure 2 illustrates the workflow used to assess GPT-5. For each dental clinical case, we applied a zero-shot prompting approach (Figure 2A [30]), in which patient information and clinical details were extracted from publications and lightly reformatted to correct formatting artifacts introduced during PDF extraction for GPT-5 input. An example prompt for a male patient aged 56 years is provided in Figure 2A.

The prompt was then submitted to GPT-5, which was asked to determine the stage and grade of periodontitis. The model’s response was collected (Figure 2B), recording only the predicted stage and grade while disregarding the diagnostic reasoning. The output was compared against the clinical diagnosis to evaluate accuracy (Figure 2C).

After all cases were processed, GPT-5’s predictions were aggregated, and performance metrics were calculated to summarize its diagnostic accuracy and Cohen κ and assess its potential use in real-world clinical settings.

### GPT-5 Performance in Periodontitis Staging and Grading

Across the 25 periodontitis cases (including the 2 borderline cases), GPT-5 achieved a staging accuracy of 68% (17/25; 95% CI 48.4%-82.8%) and a grading accuracy of 77.3% (17/22; 95% CI 56.6%-89.9%), excluding 3 (12%) cases without specified grades. The corresponding Cohen κ values were 0.454 for staging (95% CI 11.0%-75.6%) and 0.179 for grading (95% CI −15.8% to 63.8%). These findings indicate fair agreement beyond chance for staging and poor agreement for grading. The relatively wide CIs for both outcomes reflect substantial uncertainty attributable to the small sample size and class imbalance. Additional performance metrics for GPT-5, including weighted Cohen κ, balanced and stratified accuracy, and macro *F*_1_-score, are provided in Tables S2 and S3 in Multimedia Appendix 1.

**Figure 3.**
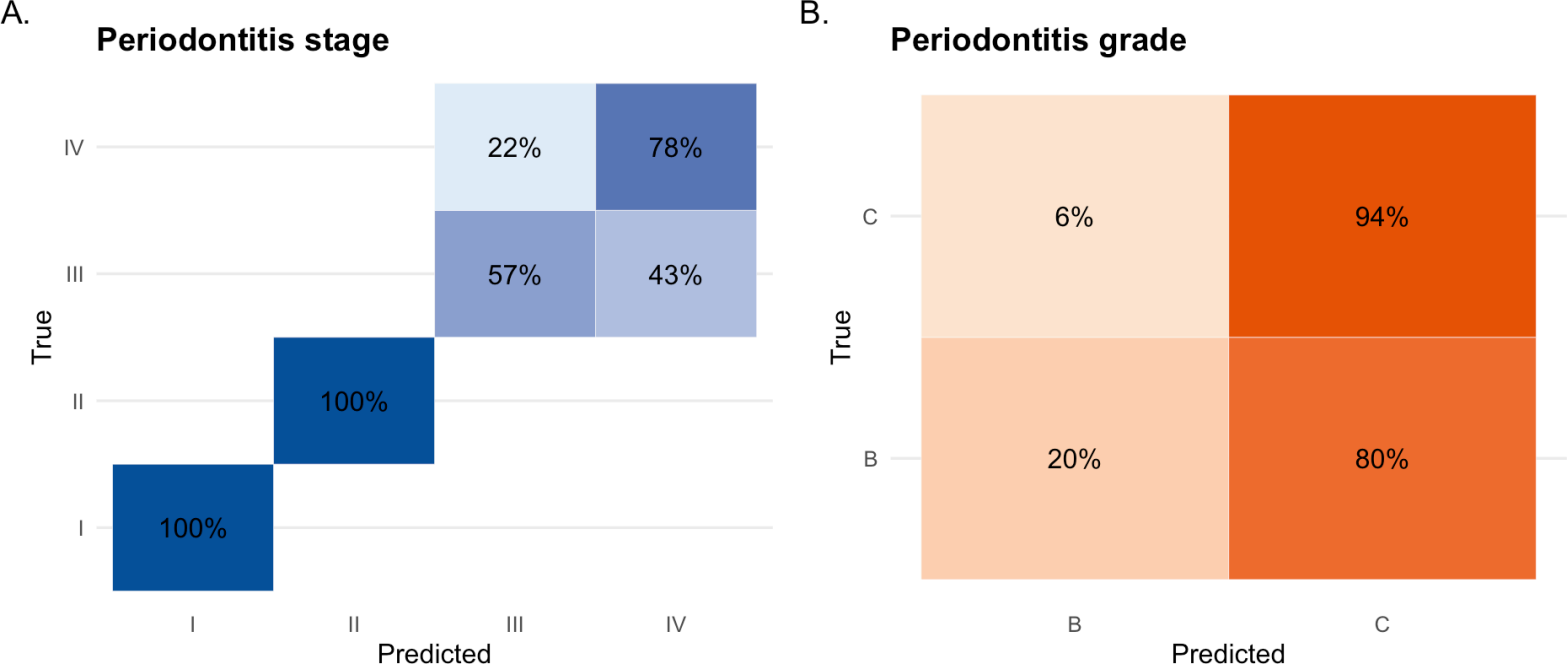
Confusion matrices of GPT-5 predictions for periodontitis staging and grading.

The confusion matrix in Figure 3A shows that all stage I and II cases were classified correctly (recall=100%), whereas recall was substantially lower for stage III (57%) and stage IV (78%). For grading, performance was markedly imbalanced, with high recall for grade C (94%) but very low recall for grade B (20%; Figure 3B). This class-dependent performance indicates that GPT-5 performs well for early-stage periodontitis and advanced disease detection but struggles to reliably distinguish intermediate disease categories.

Importantly, misclassifications exhibited nonrandom patterns: errors were confined to adjacent categories, with no stage I or II cases misclassified as stage III or IV, no stage IV cases misclassified as stage I or II (Figure 3A), and no “skipping” across nonadjacent categories for grading (Figure 3B). Because all errors were limited to adjacent stages and grades and no catastrophic misclassifications were observed, these patterns are clinically and educationally relevant.

Among the misclassified cases, 43% (6/14) of stage III cases were predicted as stage IV, while 22% (2/9) of stage IV cases were predicted as stage III (Figure 3A). For grading (Figure 3B), GPT-5 correctly classified 94% (16/17) of grade C cases, with most (4/5) grade B cases misclassified as grade C. These results suggest that GPT-5 tends to assign higher severity for both periodontitis stage and grade. GPT-5’s predictions for the 25 cases are provided in Multimedia Appendix 1.

**Figure 4.**
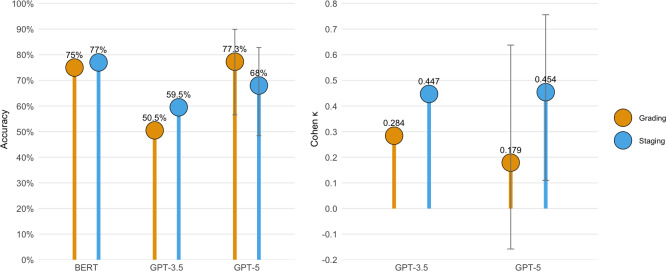
Comparison of Bidirectional Encoder Representations from Transformers (BERT), GPT-3.5, and GPT-5 performance in periodontitis staging and grading. Data are derived from Table 1. Error bars indicate 95% CIs for GPT-5, calculated from case-level data from Table S1 in Multimedia Appendix 1; CIs were not reported for BERT and GPT-3.5 in the original studies.

Table 1 and Figure 4 present contextual comparisons with 2 previous assessments. Tastan Eroglu et al [4] evaluated GPT-3.5 on 200 untreated patients and reported 59.5% accuracy in staging and 50.5% accuracy in grading—both notably lower than the performance achieved by GPT-5 on our dataset. However, this comparison should be interpreted cautiously because the datasets and evaluation protocols differ across studies. A direct head-to-head comparison was not feasible due to the unavailability of the datasets used in the previous study. The low κ values observed for both models (0.284 for GPT-3.5 and 0.179 for GPT-5) in Table 1 indicate that current LLMs have limited discriminatory ability in grading periodontitis beyond chance agreement.

**Table 1.** Recent studies of periodontitis staging and grading using textual input.

Study	Dataset^a^	Model	Accuracy (%)		Cohen κ	
			Stage	Grade	Stage	Grade
Ameli et al [31]	309 periodontal charts and clinical notes	Bidirectional Encoder Representations from Transformers (BERT)	77	75	N/A^b^	N/A
Tastan Eroglu et al [4]	200 patients with untreated periodontitis	GPT-3.5	59.5	50.5	0.447	0.284
Our result	25 dental clinical cases from public sources	GPT-5	68	77.3	0.454	0.179

a The findings from the first 2 studies were extracted from published papers, while the details of our own assessment results are provided in Multimedia Appendix 1.

b N/A: not available.

Another study, summarized in Table 1 and Figure 4, conducted by Ameli et al [31] fine-tuned a Bidirectional Encoder Representations from Transformers (BERT) model using 309 anonymized periodontal charts and corresponding clinician notes. The model was trained on 70% of the data and tested on 32 holdout cases. The fine-tuned BERT model achieved 77% accuracy in staging and 75% in grading [31]. Although these results fall within a similar range and are presented alongside GPT-5 for contextual reference, they should not be interpreted as evidence of relative model superiority, as BERT was trained and evaluated on periodontal charts and clinician notes, whereas GPT-5 and GPT-3.5 were assessed using standardized textual case descriptions without task-specific optimization.

## Discussion

### Principal Findings

This exploratory study evaluated GPT-5’s ability to perform periodontitis staging and grading using publicly available clinical case descriptions based on the 2017 World Workshop classification framework. Overall, GPT-5 demonstrated moderate diagnostic performance and fair agreement beyond chance for staging (accuracy 68%; κ=0.454), but substantially lower reliability for grading (κ=0.179). In addition, the model exhibited a tendency to overestimate disease severity. These findings suggest that while GPT-5 is capable of applying guideline-based diagnostic criteria, important limitations remain in its ability to reliably distinguish disease grades.

### Interpretation and Implications of Findings

Although this study focused narrowly on GPT-5’s performance in periodontitis staging and grading, the potential applications of LLMs extend more broadly to both clinical diagnostics and dental education. In clinical practice, LLMs could assist practitioners by consistently applying standardized staging and grading criteria, integrating charting and radiographic data, and generating preliminary assessments under appropriate clinician oversight. In education, LLMs can function as personalized learning assistants, offering structured feedback on case analyses and helping students navigate diagnostic complexity. Beyond these applications, LLM-based chatbots hold potential for reducing gaps in dental educational resources, particularly in underresourced institutions, thereby strengthening their capacity to deliver high-quality dental education and care.

However, the observed diagnostic agreement, particularly for periodontitis grading, highlights an important limitation. The low Cohen κ (κ=0.179) for grading indicates poor agreement beyond chance, suggesting that GPT-5 currently lacks sufficient reliability to accurately distinguish between periodontitis grades. Consequently, GPT-5 is not yet suitable for independent clinical grading or treatment decision-making, and its outputs should be interpreted with caution. In addition, in the absence of established minimum clinically important difference thresholds for artificial intelligence (AI)–assisted periodontitis staging and grading, κ=0.454 for staging should be interpreted cautiously and not as evidence of clinical readiness.

Looking forward, GPT-5 and other LLMs are likely to continue to improve diagnostic performance. Nevertheless, its meaningful clinical translation will hinge on overcoming current deficiencies in reliability and consistency. Beyond advancements in model development, a potential pathway toward high-stakes clinical and educational applications likely lies in the integration of LLMs with validated AI tools optimized specifically for clinical use. Such hybrid systems, which combine the precision of specialized diagnostic models with the reasoning, interpretability, and interactivity of LLMs, may provide more robust support for complex, multimodal clinical decision-making in dental care and education.

### Limitations

Our exploratory evaluation of GPT-5 relied solely on published cases, which represent a relatively limited sample. Moreover, both the small sample size and the gender imbalance within the data may disproportionately reflect more severe or well-documented presentations, potentially inflating GPT-5’s performance. In addition, publicly available case reports are often curated to highlight clear diagnostic features and may not accurately reflect the full clinical heterogeneity or noise encountered in real-world practice. As a result, such cases may underrepresent diverse patient populations and disease presentations commonly seen in routine clinical settings. Furthermore, the dataset was not constructed through a systematic review process and may, therefore, be subject to selection bias. Therefore, expanding the dataset in future studies to include larger, more diverse, and nonpublished clinical data will be essential to broaden the scope of evaluation, strengthen generalizability, and support meaningful clinical translation.

Additional methodological constraints include our use of GPT-5’s interactive interface, which does not allow modification of underlying system parameters such as temperature and may therefore restrict reproducibility at the system level. In addition, we did not evaluate model stability across alternative prompting strategies, which are known to influence LLM behavior. We restricted model inputs to textual case descriptions and did not evaluate GPT-5’s multimodal capabilities for direct radiographic interpretation. While this design was intended to isolate guideline-based reasoning, it does not reflect the full multimodal nature of periodontal diagnosis and, therefore, may not represent real-world diagnostic performance using raw clinical data. Furthermore, GPT-5 was required to provide categorical staging and grading outputs, and we did not assess its ability to recognize diagnostic uncertainty or defer ambiguous cases. Only 2 borderline or equivocal cases were included, which are insufficient to evaluate GPT-5’s performance in diagnostically challenging scenarios where clinician disagreement is common and clinical judgment plays a critical role. Finally, performance was not tested on incomplete charts or unstructured clinical notes, which may better reflect real-world variability. Therefore, future studies should incorporate more diagnostically ambiguous cases, multimodal inputs, and heterogeneous clinical documentation and systematically compare prompting strategies to better evaluate model reliability and clinical relevance.

Finally, this exploratory study was conducted with consideration of established AI reporting guidelines, including STARD or STARD-AI [28,29], DECIDE-AI [32,33], and STROBE-AI [34]. While the exploratory design and reliance on publicly available clinical cases, rather than real-time clinical data, precluded full adherence to all framework components, key principles such as transparency in data sources, model use, limitations, and reproducibility were followed. Future prospective studies using large-scale, real-world clinical data will be better positioned to fully implement these reporting standards.

### Conclusions

With the growing use of LLMs by dental and medical students, clinicians, and the general public, it is important to evaluate their performance in high-stakes diagnostic and educational settings to inform safety protocols and guide responsible applications. In this study, we assessed GPT-5’s ability to stage and grade periodontitis—tasks central to periodontal diagnosis and student training. Contextual comparisons with prior models suggested comparable or improved performance, achieving 68% staging accuracy and 77.3% grading accuracy, with a staging κ of 0.454, indicating fair agreement beyond chance. However, these findings should be interpreted cautiously, as differences in datasets, case formats, and evaluation conditions preclude direct head-to-head comparisons with prior studies.

Despite these observed performances, the low κ for grading (0.179) underscores the very limited discriminatory capacity in distinguishing periodontitis grades, indicating that GPT-5 is not yet suitable for independent clinical applications. Additionally, our results demonstrated a consistent tendency for GPT-5 to overestimate disease severity. Therefore, inappropriate reliance on model outputs could increase the risk of overtreatment or unnecessary escalation of care. This highlights the importance of human oversight and the need for future evaluations of uncertainty reporting, refusal behavior, and decision-level safeguards before any clinical integration is considered.

In conclusion, while GPT-5 demonstrated potential as a supportive tool for education and clinical exploration, it is not yet ready for autonomous use. Meaningful application in periodontal diagnosis and training will depend on substantial improvements in reliability and rigorous validation in larger, more diverse, and prospectively collected clinical datasets.

## Supplementary material

10.2196/88407 Multimedia Appendix 1 GPT-5’s performance in periodontitis staging and grading on the textual input of 25 clinical cases.

10.2196/88407 Checklist 1 STARD and STARD-AI checklist for reporting GPT-5 diagnostic accuracy.

## Abbreviations

AI: artificial intelligence
BERT: Bidirectional Encoder Representations from Transformers
LLM: large language model
MeSH: Medical Subject Headings
STARD: Standards for Reporting Diagnostic Accuracy
