# Supplementary material for: Evaluation of GPT-5 in Periodontitis Staging and Grading: Retrospective Observational Study
Source: JMIR Form Res. 2026 Apr 6;10:e88407. doi: 10.2196/88407 (PMC13052999; doi:10.2196/88407)
Supplement: Multimedia Appendix 1 [file formative-v10-e88407-s001.docx]

Appendix

1. Twenty-five periodontitis cases and GPT-5 performance

The periodontitis cases retrieved through Google and PubMed searches for this study are summarized in the table below. For each case, we copied the patient’s information, dental histories, and numerical measures of the bone loss to a text editor for minor formatting adjustments (e.g., line breaks and spacing). Then, we constructed a prompt and provided it to GPT-5 to predict periodontitis stage and grade. An example prompt and ChatGPT response are provided in the next section. The 25 cases and GPT-5 results are summarized in the Table below.

**Table S1** GPT-5 performance in periodontitis staging and grading on the textual input of 25 published cases

| **No^*^** | **Periodontitis Case** | | | | | | **GPT-5 Result^+^** | | **Source** |
| --- | --- | --- | --- | --- | --- | --- | --- | --- | --- |
|  | **Age** | **Sex** | **Stage** | **Grade** | **Alveolar Bone Loss** | **Periodontal Pocket Depth** | **Stage** | **Grade** |  |
| 1 | 5 | F | III | NA | Severe horizontal bone loss (first four primary molars) | 5 mm |  | NA | [10.5005/jp-journals-10005-1536](https://doi.org/10.5005/jp-journals-10005-1536) |
| 2 | 12 | F | III | C | Localized bone loss (first molars, incisors) | Bone density Z-score -0.74/close to critical load; no PPD value available |  |  | [10.1097/MD.0000000000035321](https://doi.org/10.1097/MD.0000000000035321) |
| 3 | 15 | F | III | C | Severe bone loss (anterior maxillary tooth) | 10 mm @ left central incisors, 8-9 mm @ central incisors | IV |  | [10.3390/diagnostics13061077](https://doi.org/10.3390/diagnostics13061077) |
| 4 | 25 | M | IV | C | Localized vertical, angular bone loss | NA | III |  | <https://bootcamp.com/blog/bootcamp-coms-perio-classification-for-the-inbde> |
| 5 | 25 | M | IV | C | Generalized horizontal and vertical bone loss with loss of periapical bone | 8–10 mm |  |  | [10.4103/ccd.ccd_336_19](https://doi.org/10.4103/ccd.ccd_336_19) |
| 6 | 32 | F | III | B | Extensive vertical bone loss and bone resorption @ left 1^st^-2^nd^ maxillary molar, right 2^nd^ mandibular molar | 4–7 mm |  | C | [10.1186/s13256-023-03751-1](https://doi.org/10.1186/s13256-023-03751-1) |
| 7 | 35 | F | III | C | Severe alveolar bone loss @ right upper lateral incision and left canine | 4.3 mm,  40.5% @ 7mm | IV |  | [10.12998/wjcc.v9.i21.6110](https://doi.org/10.12998/wjcc.v9.i21.6110) |
| 8 | 37 | F | III | C | Localized bone loss; vertical bone loss up to 50% | 4–7 mm |  |  | [10.1038/sj.bdj.2019.93](https://doi.org/10.1038/sj.bdj.2019.93) |
| 9 | 37 | F | III | B | ~50% interdental bone loss | ~7 mm |  | C | [10.3390/jcm13051451](https://doi.org/10.3390/jcm13051451) |
| 10 | 37 | M | III | NA | Generalized horizontal and vertical bone resorption (#22, 36, 37, 46) | NA | IV | NA | [10.7759/cureus.73336](https://doi.org/10.7759/cureus.73336) |
| 11 | 41 | M | III | C | Alveolar bone loss with extension to root apex (#37, #47); angular bone defects (#17, 24, 37, 47); furcation radiolucency (#17, 27, 37, 47) | 4–5 mm (48.8%); ≥6 mm (23.8%) | IV |  | [10.7759/cureus.85449](https://doi.org/10.7759/cureus.85449) |
| 12 | 42 | F | IV | NA | Generalized bone destruction @ maxillary and mandibular teeth | 5–8 mm |  | NA | [10.4103/jid.jid_33_21](https://doi.org/10.4103/jid.jid_33_21) |
| 13 | 45 | F | III | C | Full mouth alveolar bone loss. Middle ½ to apical ⅓ of root; #26–27 apical absorbed in apical region; periapical radiolucency #25. | ≥4 mm (44.6%); ≥6 mm (15.5%) | IV |  | [10.1097/MD.0000000000042037](https://doi.org/10.1097/MD.0000000000042037) |
| 14 | 47 | F | II | B | Max Bone loss 30% #15, 27 mesial); exceeding 15% of root length confined to coronal third (#17, 16,15, 26, 27); % Bone loss > ½ age/years (30>23.5) | Max 6 mm |  |  | <https://discovery.ucl.ac.uk/id/eprint/10069989/1/Needleman_case_report2_v03.pdf> |
| 15 | 47 | F | IV | C | Full mouth alveolar bone loss @ middle ½ to apical ⅓ of root; apical resorption (#22, 31) | >4 mm (>50%) |  |  | [10.12998/wjcc.v10.i7.2229](https://doi.org/10.12998/wjcc.v10.i7.2229) |
| 16 | 50 | M | III | B | Grade 1-2 furcation @ upper molars; no imaging available | 2–5 mm anterior; 2–8 mm posterior |  | C | <https://deepblue.lib.umich.edu/bitstream/handle/2027.42/162782/cap10095.pdf> |
| 17 | 54 | F | III | C | Vertical bone defects @ molar region | ≥4 mm (30.4%) |  |  | [10.2209/tdcpublication.2022-0017](https://doi.org/10.2209/tdcpublication.2022-0017) |
| 18 | 55 | F | IV | C | Extensive alveolar bone resorption extending to the root apex and attachment loss in most maxillary/mandibular molars | NA |  |  | [10.1186/s13256-022-03703-1](https://doi.org/10.1186/s13256-022-03703-1) |
| 19 | 56 | M | IV | C | Class II furcation; multiple teeth | >5 mm widespread |  |  | <https://bootcamp.com/blog/bootcamp-coms-perio-classification-for-the-inbde> |
| 20 | 56 | F | III | C | Angular bone resorption (#18, 48); Horizontal additional regions; % Bone loss at age 48= 1.07 | ≥4 mm (34.0%) |  |  | [10.2209/tdcpublication.2023-0018](https://doi.org/10.2209/tdcpublication.2023-0018) |
| 21 | 59 | F | IV | C | Bone loss > 30% of affected teeth; Max extension to apical ½ root, 70% bone loss on #14 | ≤4 mm |  |  | [10.1038/s41415-019-0143-8](https://doi.org/10.1038/s41415-019-0143-8) |
| 22 | 62 | F | IV | C | Furcation of all molars; apical/external root resorption of upper molar/lower right 2^nd^ molar | Severe generalized; no PPD value available |  |  | [10.1002/cap.10232](https://doi.org/10.1002/cap.10232) |
| 23 | 63 | M | I | C | Distance from CEJ to alveolar bone crest = 4mm; root length 12 mm (#29); 4-2 mm/12mm= 16.7% bone loss | NA |  |  | <https://www.scribd.com/document/862481006/> |
| 24 | 64 | F | IV | C | Generalized bone loss extending to the coronal 1/3 of the root with 8 vertical defects (#2, 4, 4, 14, 15, 19, 21, 30) | NA | III | B | [10.1002/cap.10129](https://doi.org/10.1002/cap.10129) |
| 25 | 66 | F | III | B | Generalized bone loss extending to the mid 1/3 root with 3 vertical defects (#13, 14, 26) | General interproximal attachment 5 mm (30%); no PPD value available | IV | C | [10.1002/cap.10129](https://doi.org/10.1002/cap.10129) |
| **Number of correction predictions/total cases** | | | | | | | 17/25 | 17/22 |  |
| **Accuracy** | | | | | | | 68.0% | 77.3% |  |

**Note:** ^*^The last two cases fall into gray zones where categorizing the patient requires clinical judgment.

^+^The blank spaces in the two columns represent correct predictions of GPT-5.

2. Supplementary analyses of GPT-5 diagnostic performance

Weighted Cohen’s κ (quadratic weighting for staging and linear weighting for grading) was calculated from Table S1 using the kappa2() function in the R package irr (version 0.84.1), to account for the ordinal structure of periodontitis staging (I–IV) and grading (A–C). Ninety-five percent confidence intervals (CI) were estimated using nonparametric bootstrap resampling (2,000 iterations).

To address potential class imbalance, we also calculated balanced accuracy and macro-averaged F1 scores. Balanced accuracy was defined as the mean recall (sensitivity) across all classes, thereby weighting each stage or grade equally, regardless of class frequency. Macro F1 was computed as the unweighted mean of the class-specific F1 scores, where the F1 score for each class is the harmonic mean of precision and recall.

All analyses were conducted in R (version 4.3.3). The resulting agreement estimates, balanced accuracy, and macro-averaged F1 scores are presented in Table S2 below. The weighted κ for staging (0.706) in Table S2 was notably higher than the corresponding unweighted κ (0.454) in the paper, suggesting that most staging disagreements occurred between adjacent categories and therefore reflected partial rather than substantial discordance.

**Table S2** Weighted κ, balanced accuracy, and macro-averaged F1 scores for GPT-5

| ****Diagnostic task**** | ****Weighted Cohen’s κ**** | ****95% CI**** | ****Balanced accuracy**** | ****Macro F1**** |
| --- | --- | --- | --- | --- |
| Periodontitis staging | 0.706 | 0.157-0.884 | 0.837 | 0.826 |
| Periodontitis grading | 0.179 | -0.158-0.645 | 0.571 | 0.575 |

**Note**: 95% CI were estimated using bootstrap resampling (2,000 iterations). Invalid resamples yielding undefined κ values were excluded.

Table S3 presents GPT-5’s diagnostic accuracy stratified by sex and age subgroups. Fisher’s exact tests did not demonstrate statistically significant differences across sex or age categories (p > 0.05).

**Table S3** Stratified performance of GPT-5 in periodontitis staging and grading

| **Diagnostic task** | **Subgroup** | **n** | **Stratified accuracy** | **95% CI** |
| --- | --- | --- | --- | --- |
| Staging | Female | 18 | 0.722 | 0.491–0.875 |
|  | Male | 7 | 0.571 | 0.250–0.842 |
| Grading | Female | 16 | 0.750 | 0.505–0.898 |
|  | Male | 6 | 0.833 | 0.436–0.970 |
| Staging | <40 | 10 | 0.6 | 0.313–0.832 |
|  | 40–59 | 11 | 0.818 | 0.523–0.949 |
|  | ≥60 | 4 | 0.5 | 0.150–0.850 |
| Grading | <40 | 8 | 0.75 | 0.409–0.929 |
|  | 40–59 | 10 | 0.9 | 0.596–0.982 |
|  | ≥60 | 4 | 0.5 | 0.150–0.850 |

3. Zero-shot prompting to produce the results in Table S1.

To generate the results shown in Table S1 above, we employed a zero-shot prompting approach, providing GPT solely with textual descriptions of dental patients and asking it to determine the stage and grade of the periodontitis without offering any examples or prior instruction. As an illustration of this method, the following is the full prompt used for case 19 in Table S1:

**3.1 A full prompt for ChatGPT:**

Can you help determine periodontitis stage and grade of this patient:

Male, 56 years old

Chief Complaint: "My teeth are getting loose and falling out."

Background and/or Patient History:

Diabetes (last HbA1c 8.1%)

Hypertension

Last dental visit 2 years ago

Medications:

Hydrochlorothiazide (Microzide®)

Metformin (Glumetza®)

Multivitamin

Current Findings: Generalized bleeding on probing, widespread probing depths greater than 5mm, class II furcation involvement in multiple teeth, multiple teeth with grade Il mobility, missing teeth 1, 4,13, 16, 17, 18, 20, 29, 30, and 32.

**3.2 ChatGPT’s response to the prompt above:**

Let’s go step-by-step, following the 2017 World Workshop on the Classification of Periodontal and Peri‐Implant Diseases and Conditions criteria.

1. Establish that this is periodontitis

- Evidence of interdental CAL: Yes (widespread probing depths >5 mm, mobility, furcations, tooth loss due to periodontal breakdown is very likely).
- Chronicity and progression: Progressive tooth loss and mobility reported.
- Not due to non-periodontal causes: Findings are consistent with periodontal disease, not trauma or recession alone.
  ✅ → Diagnosis confirmed: Periodontitis.

2. Determine Stage (Severity + Complexity)

A. Severity (based on CAL, RBL, tooth loss)

- Missing teeth: #1, 4, 13, 16, 17, 18, 20, 29, 30, 32 (10 missing in total).
- Need to determine how many missing due to periodontitis. Given the pattern and mobility, at least some posterior tooth loss likely due to perio.
- Stage definitions for severity:
  - Stage III: Interdental CAL ≥5 mm, RBL extending to middle third of root or beyond, and ≤4 teeth lost due to periodontitis.
  - Stage IV: Same CAL/RBL but ≥5 teeth lost due to periodontitis.

Given the loose teeth, multiple molars missing, and likely ≥5 teeth lost from periodontitis, severity meets Stage IV.

B. Complexity

- Probing depths >5 mm generalized.
- Furcation involvement (Class II in multiple teeth).
- Grade II mobility in multiple teeth.
- Reduced masticatory function due to missing teeth.
  This also supports Stage IV complexity.

✅ Stage: IV

3. Determine Grade (Rate of progression + risk factors)

Primary criterion: Evidence of progression

- Tooth loss and bone loss pattern indicate rapid progression relative to age.

Indirect evidence:

- HbA1c 8.1% → poorly controlled diabetes → strong risk modifier.
- No smoking history noted.

Grading framework:

- Grade B: Moderate progression, RBL ≈ age.
- Grade C: Rapid progression, RBL exceeds expected for age, and/or strong risk factors (poorly controlled diabetes, heavy smoking).

Poorly controlled diabetes alone moves this patient to Grade C, even without calculating exact RBL/age ratio.

✅ Grade: C

Final Classification

Periodontitis, Stage IV, Grade C

- Stage IV: Severe CAL, ≥5 teeth lost due to periodontitis, masticatory dysfunction, deep pockets, furcations, mobility.
- Grade C: Rapid progression with major systemic risk factor (poorly controlled diabetes).
