# Supplementary material for: Evaluation of GPT-5 in Periodontitis Staging and Grading: Retrospective Observational Study
Source: JMIR Form Res. 2026 Apr 6;10:e88407. doi: 10.2196/88407 (PMC13052999; doi:10.2196/88407)
Supplement: Checklist 1 [file formative-v10-e88407-s002.pdf]

Section & Topic	No	Item	Reported on page #
**TITLE OR ABSTRACT**			
	**1**	Identification as a study of diagnostic accuracy using at least one measure of accuracy (such as sensitivity, specificity, predictive values, or AUC)	Accuracy & Cohen's κ, Abstract (p. 1)
**ABSTRACT**			
	**2**	Structured summary of study design, methods, results, and conclusions (for specific guidance, see STARD for Abstracts)	Abstract (p. 1)
**INTRODUCTION**			
	**3**	Scientific and clinical background, including the intended use and clinical role of the index test	Introduction (p. 1-2)
	**4**	Study objectives and hypotheses	Introduction (p. 2)
**METHODS**			
Study design	**5**	Whether data collection was planned before the index test and reference standard were performed (prospective study) or after (retrospective study)	Methods – Case Identification (p.2)
Participants	**6**	Eligibility criteria	Methods – Case Identification (p.2)
	**7**	On what basis potentially eligible participants were identified (such as symptoms, results from previous tests, inclusion in registry)	Not applicable (published cases)
	**8**	Where and when potentially eligible participants were identified (setting, location and dates)	Not applicable
	**9**	Whether participants formed a consecutive, random or convenience series	Not applicable
Test methods	**10a**	Index test, in sufficient detail to allow replication	Methods – GPT-5 Prompting (p. 3)
	**10b**	Reference standard, in sufficient detail to allow replication	Published reference diagnoses (p. 2)
	**11**	Rationale for choosing the reference standard (if alternatives exist)	Methods (p. 2)
	**12a**	Definition of and rationale for test positivity cut-offs or result categories of the index test, distinguishing pre-specified from exploratory	Periodontitis Stage I-V & Grade A-C (p. 3)
	**12b**	Definition of and rationale for test positivity cut-offs or result categories of the reference standard, distinguishing pre-specified from exploratory	2017 AAP/EFP (Methods, p. 2)
	**13a**	Whether clinical information and reference standard results were available to the performers/readers of the index test	GPT evaluated cases independently (p. 3)
	**13b**	Whether clinical information and index test results were available to the assessors of the reference standard	Published reference diagnoses (p. 4)
Analysis	**14**	Methods for estimating or comparing measures of diagnostic accuracy	Accuracy and κ (p. 3)
	**15**	How indeterminate index test or reference standard results were handled	Results (p. 4)
	**16**	How missing data on the index test and reference standard were handled	Incomplete cases excluded (p. 4)
	**17**	Any analyses of variability in diagnostic accuracy, distinguishing pre-specified from exploratory	Methods (p. 3)
	**18**	Intended sample size and how it was determined	Methods (p. 2)
**RESULTS**			
Participants	**19**	Flow of participants, using a diagram	Figure 1 (p. 3)
	**20**	Baseline demographic and clinical characteristics of participants	Case description(p. 4)
	**21a**	Distribution of severity of disease in those with the target condition	Case description(p. 4)
	**21b**	Distribution of alternative diagnoses in those without the target condition	Not applicable
	**22**	Time interval and any clinical interventions between index test and reference standard	Not applicable
Test results	**23**	Cross tabulation of the index test results (or their distribution) by the results of the reference standard	Figure 3 (p. 5)
	**24**	Estimates of diagnostic accuracy and their precision (such as 95% confidence intervals)	Results (p. 4-5)
	**25**	Any adverse events from performing the index test or the reference standard	Not applicable
**DISCUSSION**			
	**26**	Study limitations, including sources of potential bias, statistical uncertainty, and generalisability	Discussion – Limitations (p. 7)
	**27**	Implications for practice, including the intended use and clinical role of the index test	Interpretation (p. 6)
**OTHER INFORMATION**			
	**28**	Registration number and name of registry	Not applicable
	**29**	Where the full study protocol can be accessed	Not applicable
	**30**	Sources of funding and other support; role of funders	Funding (p. 7-8)

CC BY SA
